# Leaf color as an indicator of the accumulation pattern of pharmaco-nutritional compounds in *Lithocarpus litseifolius* spring foliage

**DOI:** 10.3389/fpls.2025.1726934

**Published:** 2025-12-10

**Authors:** Pengxia Zhao, Zhiling Yang, Zifeng Tan, Yixun Yang, Zhaoxia Tian, Xu Yang

**Affiliations:** 1 Research Institute of Subtropical Forestry, Chinese Academy of Forestry, Hangzhou, China; 2 College of Horticulture, Hebei Agricultural University, Baoding, China

**Keywords:** *Lithocarpus litseifolius*, spring foliage, developmental stage, pharmaco-nutritional components, leaf color, correlation analysis

## Abstract

*Lithocarpus litseifolius* leaves are rich in pharmaco-nutritional compounds such as phlorizin and trilobatin, and their spring foliage exhibits vibrant colors. However, the relationship between leaf color and the levels of these compounds remains unexplored. This study investigated the growth patterns of naturally variegated *Lithocarpus litseifolius* and measured the pharmaco-nutritional component content using UV-Vis and HPLC, along with the correlation between these components and leaf color. The results showed that spring foliage followed an S-shaped growth curve, while total flavonoid content of the pharmaco-nutritional components exhibited a multi-peak accumulation pattern, initially increasing before declining. Trilobatin levels peaked on April 3 and April 8, whereas phlorizin reached its highest level on May 31. The flavonoid composition of different-colored leaves varied consistently during leaf development, with reddish-brown (RBL) and orange (OL) leaves containing the highest levels. Correlation analysis revealed a significant positive relationship between redness (a*) and flavonoid content—higher anthocyanin levels corresponded to greater flavonoid accumulation. Thus, the optimal period for tea preparation is early April, while late May, at full leaf maturity, is ideal for pharmaceutical harvest. The reddish-brown (RBL) and orange (OL) leaves, having the highest flavonoid content, are best suited for breeding. Therefore, the strong correlation between leaf color and flavonoid composition suggests that color can serve as a morphological indicator for assessing flavonoid accumulation.

## 1 Introduction

Plant phenotypes are stable expressions of shape, structure, size, and color, determined by both genotype and environment (Pan, 2015). Phenotypic traits serve as important indicators of adaptation, selection, survival, and evolution, reflecting variation within plant species (Guo et al., 2023; Dwivedi et al., 2016; Gould et al., 2010). Among these traits, leaf color is a key component, with changes often indicating gene-level alterations in pigment metabolism. Plants are influenced by various factors, including light, temperature, water, and exogenous hormones, which stimulate the expression of transcription factors and structural genes such as *HY5, PIFS*, and *DELLA*. Chromatin modifications (e.g., methylation and acetylation), along with post-transcriptional epigenetic regulation by microRNAs (miRNAs) and long non-coding RNAs (lncRNAs), have been shown to regulate genes involved in the biosynthesis of chlorophylls, carotenoids, and anthocyanins. These regulatory mechanisms directly or indirectly influence leaf color formation (Huang et al., 2019; Zhang et al., 2021; Jing and Lin, 2020; Liang and He, 2018). In medicinal plants, color variation often reflects differences in bioactive components. Du et al. (2017) found that seven red-variegated *Eucommia ulmoides* leaf types contained significantly higher levels of chlorogenic acid, geniposide, and total flavonoids compared to green leaves. Similarly, Guan et al. (2024) found that purple-leaf perilla (*Perilla frutescens* var. *crispa* f. *purpurea*) had significantly higher total flavonoid and phenolic content, anthocyanins, and antioxidant capacity than its green-leaf counterpart (*Perilla frutescens* var. *crispa* f. *viridis*).

The developmental stage of plant organs also affects secondary metabolite accumulation, influencing optimal harvesting times (Xu et al., 2018; Cheng et al., 2024). For instance, wild *Scutellaria barbata* D. Don shows the highest baicalin content in aerial parts during spring and in roots during autumn (Cheng et al., 2024). In *Zingiber mioga* (Thunb.) Roscoe, the concentrations of anthocyanins, proanthocyanidins, flavonols, and flavonoids in flower buds increase with growth and peak at maturity (Liu et al., 2023). Similarly, Cui et al. (2023) found that young leaves of *Osmanthus fragrans* ‘Qiannan Guifei’ had the highest anthocyanin (Cyanidin-3-O-rutinoside) content, while the flavonoid (apigenin-7-O-glucoside) and flavonol (quercetin-3-O-rutinoside), kaempferol-3-rutinoside, and kaempferol-7-O-rutinoside levels decreased significantly with leaf development. Therefore, investigating flavonoid accumulation patterns and their dynamic changes in differently colored leaves can help optimise harvesting periods.

*Lithocarpus litseifolius* (Hance) Chun is an evergreen tree widely distributed south of the Qinling Mountains (Cheng et al., 2016). Its leaves contain various flavonoid compounds, including dihydrochalcone glycosides—phlorizin, trilobatin, and phloretin (Wang et al., 2022)—which have demonstrated therapeutic effects against acute liver injury (Zuo et al., 2014), diabetes mellitus (Dong et al., 2012), cardiovascular disease (Shang et al., 2022), and obesity (Shen et al., 2021). The plant’s medicinal and nutritional value has been extensively studied, with research focusing on the extraction process (Sun et al., 2015; Shang et al., 2020), detection method (Tian et al., 2022), and pharmacological efficacy (Shang et al., 2022). Current studies on *L. litseifolius* germplasm primarily address wild resource collection and preservation, as well as the variation in medicinal compound content (e.g., phlorizin, trilobatin) across seed sources and batches (Yang et al., 2021; Yang et al., 2025; Wang et al., 2016; Wang K. et al.,2019). However, the accumulation patterns of active ingredients in young leaves of *L. litseifolius* remains poorly documented, and the potential link between leaf color and compound accumulation is yet to be fully explored.

In this study, we investigated the temporal dynamics of bioactive compounds in spring leaves of *L. litseifolius* across developmental stages and compared content among plants with different leaf colors. The aim was to clarify accumulation patterns of medicinal and edible compounds and assess whether leaf color could serve as a visual indicator of active compound levels. Current studies will offer a theoretical basis for identifying the optimal harvest time and developing a visual assessment system for selecting high-quality germplasm.

## 2 Materials and methods

### 2.1 Experimental materials and sample collection

**Figure 1:**
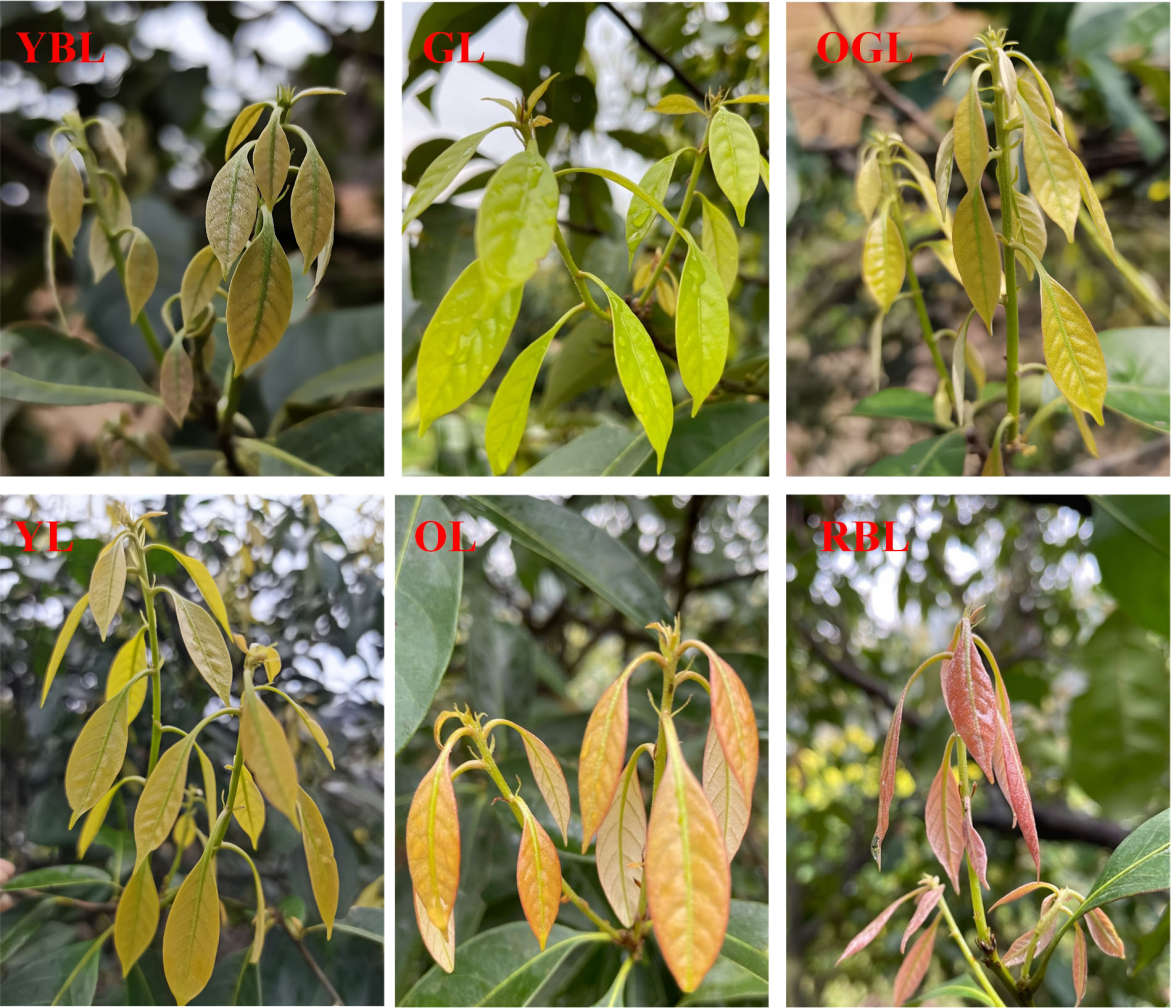
Six leaf colors of *Lithocarpus litseifolius*.

The experimental materials were obtained from natural variant plants of the seed source in Jiangshan City, Zhejiang Province, at the *L. litseifolius* germplasm resource nursery (latitude 28°22′54″N, longitude 118°30′45″E, altitude 315.2 m). All plants were cultivated for the same duration under uniform growth conditions, with adequate light and controlled artificial interventions. After three years of observation and *ex situ* cultivation at three sites—Jiangshan (Zhejiang), Fuyang (Zhejiang), and Xupu (Hunan)—the spring-emerging young leaves of *L. litseifolius* were found to exhibit six consistently distinct and stable colors. As the leaves matured, their surfaces became leathery and their pigmentation gradually deepened to dark green. These colors were compared with the RHS Plant Color Chart and designated as yellow-brown (YBL), light green (GL), yellow-green (OGL), yellow (YL), orange (OL), and red-brown (RBL) (**Figure 1**).

In March 2024, on the first day after leaf-bud unfolding, 30 identification tags were placed on three plants displaying the same leaf color. For each color type, individual plants with uniform genetic backgrounds were selected as biological replicates. Spring branch elongation and leaf length and width were measured every 5–8 days until growth stabilized. At each observation, 10 newly emerged spring shoots near each tag were collected, providing 30 shoots per color type. Shoots were separated into branches and leaves, weighed, and oven-dried at 105°C for 30 min. The temperature was then reduced to 65°C until a constant weight was reached. The branches were reweighed, crushed, and stored. Simultaneously, leaf samples were collected during the tea-picking period (24 March to 3 April). Some were used for color parameter analysis, while others were frozen in liquid nitrogen for anthocyanin content determination.

### 2.2 Measurement indicators and methods

#### 2.2.1 Measurement of color values

The brightness (L*), redness (a*), and yellowness (b*) values of young leaves of *L. litseifolius* were determined using a colorimeter (CHROMA METER CR-400, KONICA MINOLTA).

#### 2.2.2 Determination of pigment and active ingredient

##### 2.2.2.1 Anthocyanin determination

The anthocyanin content was measured using the ultraviolet spectrophotometry method described by Luo et al. (2017).

##### 2.2.2.2 Preparation of the test solution

A 0.200–0.300 g sample of *L. litseifolius* powder was weighed into a 50 mL centrifuge tube, mixed with 10 mL of 80% ethanol, and sonicated for 10 min. The mixture was centrifuged (8000 rpm, 4°C, 10 min), and the extract transferred to a 25 mL stoppered test tube. The residue was extracted again and combined with the first extract. An 80% ethanol solution was added, and the volume was adjusted to 25 mL to obtain the extract for subsequent analyses. Before liquid chromatography, samples were filtered through a 0.22 μm organic membrane.

##### 2.2.2.3 Determination of total flavonoids

Total flavonoid content was determined by a spectrophotometric method. The total flavonoid content was determined using the NaNO_2_-Al(NO_3_)_3_-NaOH colorimetric method with quercetin as a standard. A 250 μL of the extract was transferred into a 10 mL glass tube, followed by the addition of 300 μL of 5% NaNO_2_. After being allowed to stand for 6 min, 300 μL of 10% Al(NO_3_)_3_ was added and incubated for another 6 min. Subsequently, 2 mL of 1 M NaOH was added, and the mixture was diluted to 10 mL with 30% ethanol. The solution was mixed thoroughly at room temperature for 30 min, and absorbance at 510 nm was measured using a spectrophotometer. The standard curve was plotted with mass (mg) on the x-axis and absorbance on the y-axis: A = 0.557X-0.0051 (R^2^ = 0.9945).

##### 2.2.2.4 Determination of phlorizin and trilobatin

Phlorizin and trilobatin were extracted by high-performance liquid chromatography (HPLC), Tian et al. (2022) using a Water Atlantis T3 column (250 × 4.6 mm, 5 µm). The mobile phase consisted of methanol (phase A) and water (phase B) in a 52:48 (v/v) ratio; Flow rate was 1.0 mL/min, injection volume 2 µL, detection wavelength 285 nm, and column temperature 30°C.

Investigation of Linear Relationship: A 100 μL aliquot of each trilobatin and phlorizin standard solution was transferred into a 1 mL glass vial, followed by the addition of 800 μL methanol to obtain a 100 μg/mL stock solution. This standard solution was subsequently diluted to 100, 80, 40, 20, 10, and 5 μg/mL. Each dilution was then injected for analysis. Linear regression was performed with peak area plotted on the y-axis and concentration on the x-axis. Both compounds exhibited strong linearity within 5–100 μg/mL. The trilobatin calibration curve was y = 528693x – 779144 (R² = 0.912), and the phlorizin curve was y = 434841x – 640787 (R² = 0.9254).

Precision test: The test solution was injected six times and relative peak areas were recorded. The relative standard deviation (RSD) ranged from 0.227% to 4.079%, remaining below 5% and indicating acceptable precision.

Stability and repeatability test: The same batch of test solutions was injected at 0, 2, 4, 6, 8, 12, and 24 hours. The RSD ranged from 0.897% to 2.286% (remaining below 5%), Thereby confirming 24-hour stability and good repeatability.

Standard addition recovery test: Six portions of sample (0.25 g each, with known analyte content) were accurately weighed, spiked with standards equivalent to 50% of the native content, and analyzed. The average recovery rates for trilobatin and phlorizin were 96.33% and 96.85%, with RSDs of 1.30% and 1.96%, respectively, both < 5%.

### 2.3 Statistical analysis

Developmental chromaticity, growth indices, and active-ingredient content for the six leaf types were recorded using Excel 2016. One-way ANOVA of leaf phenotypes and components was performed in IBM SPSS Statistics 25.0, with multiple comparisons conducted using Duncan’s method. Data are expressed as mean ± standard deviation (SD) (n = 3). Pearson’s correlation analysis was used to assess relationships between leaf color and the content of medicinal and edible active ingredients. Bar graphs and trend curves were generated using Origin 2021.

## 3 Results

### 3.1 Analysis of color and anthocyanin differences in *L. litseifolius* leaves

The spring-grown young leaves of *L. litseifolius* showed marked color differences (**Table 1**). The L* values of YL leaves were significantly higher than those of other color types, while the a* values of RBL leaves were significantly higher and b* values lower. These color indices corresponded closely with their visible phenotypes. The anthocyanin content was subsequently measured across leaf types, revealing that RBL leaves had significantly higher levels than the others, with content decreasing in the order: RBL > YBL > OL > OGL > YL > GL.

**Table 1:** Chromaticity value and anthocyanin content of leaves of *Lithocarpus litseifolius*.

Materials	RHSCC values	L*	a*	b*	Anthocyanin (mg/100g)
YBL	N199B	41.32 ± 0.17 d	1.55 ± 0.20 c	19.83 ± 0.11 c	1.009 ± 0.0045 b
GL	146B	50.57 ± 1.75 b	-15.54 ± 0.55 f	37.19 ± 2.99 a	0.094 ± 0.013 f
OGL	152A	51.11 ± 1.93 b	-11.34 ± 0.63 e	36.42 ± 1.34 a	0.326 ± 0.019 d
YL	153A	54.50 ± 1.08 a	-7.03 ± 0.86 d	35.11 ± 1.40 a	0.237 ± 0.003 e
OL	21A	48.72 ± 2.31 c	2.55 ± 0.45 b	29.83 ± 2.10 b	0.661 ± 0.016 c
RBL	177A	42.82 ± 0.77 d	8.10 ± 0.88 a	17.20 ± 0.97 d	1.275 ± 0.043 a

Different lowercase letters indicate significant differences between leaf colors in the same period (P < 0.05).

### 3.2 Analysis of growth dynamics of *L. litseifolius* with different leaf colors

**Figure 2:**
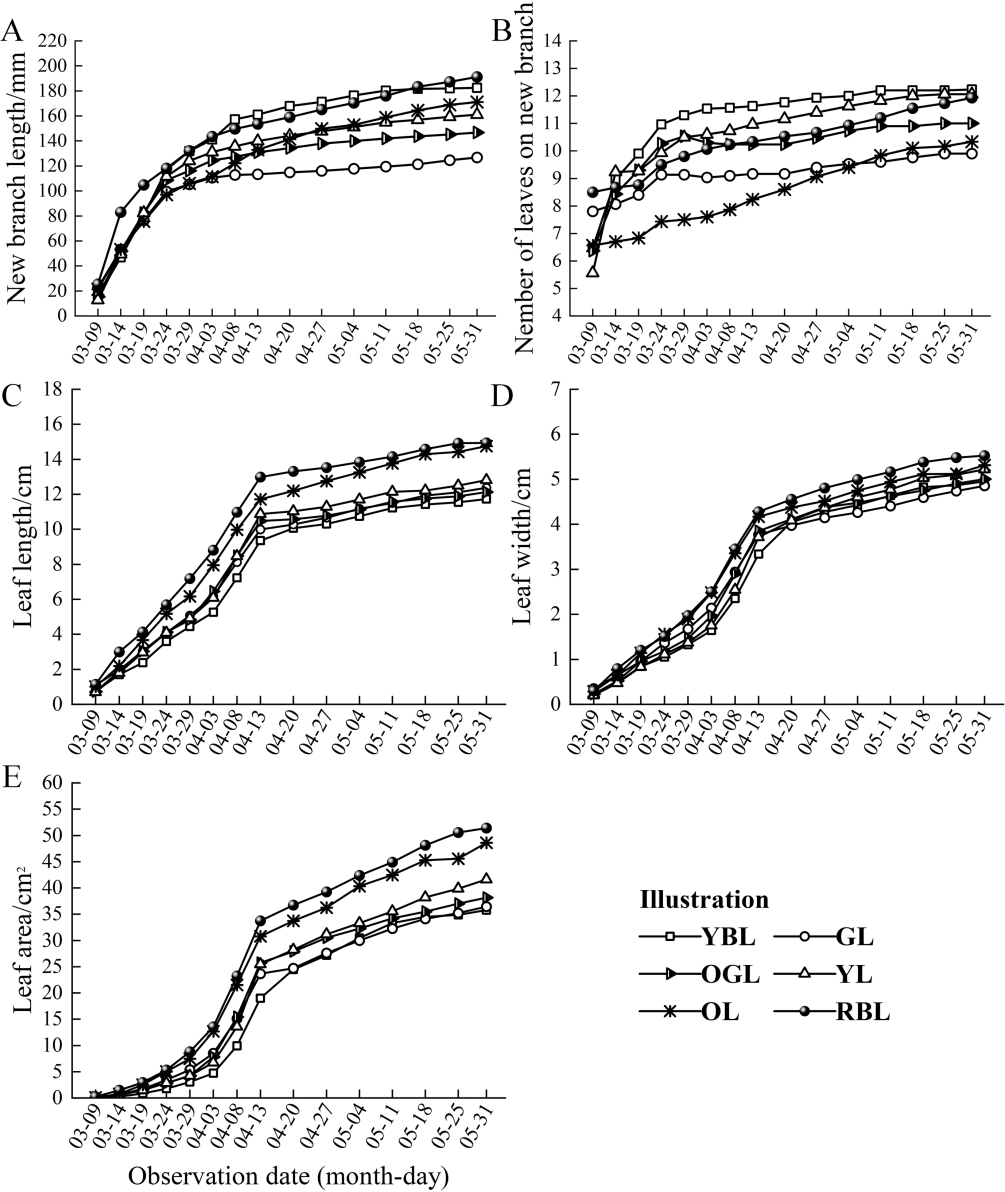
Number of leaves on branches and leaf growth dynamics of *Lithocarpus litseifolius* plants of different leaf colors.

The growth of *L. litseifolius* followed an S-shaped curve, with consistent trends in branch elongation and increases in leaf length, width and area. From 9 to 24 March, new branches expanded rapidly, with a sharp rise in leaf numbers. By 24 March, YBL, GL, and OGL leaves had reached 89.70–93.36% of their total number for the entire developmental period. New branch and leaf growth slowed thereafter, ending in late spring, with an average branch length of 163.26 mm across six leaf types by 31 May. New branch length followed the order: RBL > YBL > OGL > GL, with RBL showing the greatest and GL the least growth—only 66.31% of RBL’s length. The average number of leaves per shoot was 11.24, with YBL having the most (12.23) and GL the fewest (9.90) (**Figures 2A, B**). In early March, new branches and leaves began emerging, and leaf biomass increased rapidly. This marked a phase of accelerated leaf growth, with significant increases in leaf length, width, and area. By 13 April, leaf length grew from 0.91 cm to 10.90 cm, width from 0.27 cm to 3.85 cm, and area from 0.10 cm2 to 26.40 cm2. The leaf growth rates rose from early to mid-April, then leaf length and width increased slowly. By the end of spring, RBL exhibited the most pronounced growth, with leaf length, width, and area reaching 14.94 cm, 5.53 cm, and 51.41 cm2, respectively (**Figures 2C-E**). (**Supplementary Table 1**).

### 3.3 Active constituents of medicinal food during leaf development of different-colored *L. litseifolius* leaves

#### 3.3.1 Total flavonoids content in leaves of different leaf colors of *L. litseifolius*

**Figure 3:**
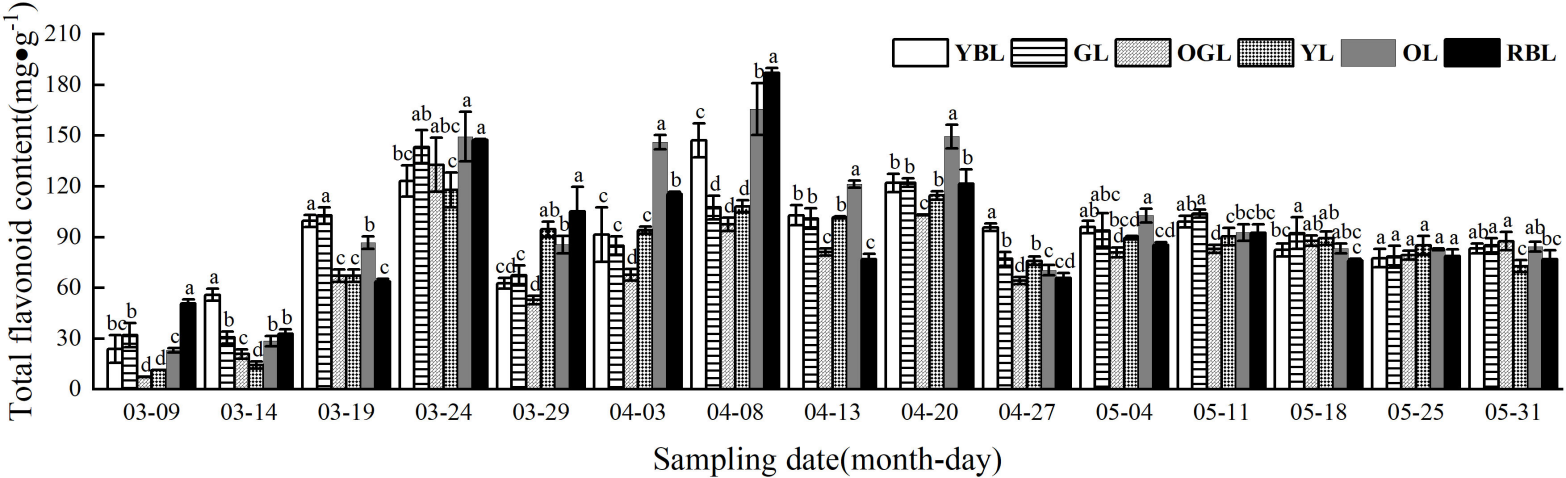
Total leaf flavonoid content of *Lithocarpus litseifolius* plants at developmental stage for different leaf colors. Different letters indicate significant differences at 0.05 level. The same below.

The total flavonoid content of *L. litseifolius* leaves was lowest at the start of leaf bud germination, subsequently increasing gradually with leaf development. As demonstrated in **Figure 3**, three accumulation peaks were observed to form on the 24th of March, 8th of April, and 20th of April, respectively. Among these, the accumulation levels of GL (143.28 ± 9.89 mg/g), OGL (132.78 ± 15.88 mg/g), and YL (117.90 ± 10.29 mg/g) reached their highest levels during the developmental period at the first peak, though these values remained lower than those of OL and RBL. YBL (147.18 ± 10.15 mg/g), OL (165.65 ± 15.37 mg/g), and RBL (186.96 ± 2.99 mg/g) exhibited the highest flavonoid content during the second accumulation peak, significantly exceeding those of GL, OGL, and YL. RBL showed the highest content, at 1.74, 1.92, and 1.73 times the levels of GL, OGL, and YL, respectively. By 13 April, leaf growth was essentially complete, young leaves had turned green, became thicker and leathery, and the flavonoid content tended to stabilize (**Figure 3**).

#### 3.3.2 Phlorizin content in leaves of different leaf colors of *L. litseifolius*

**Figure 4:**
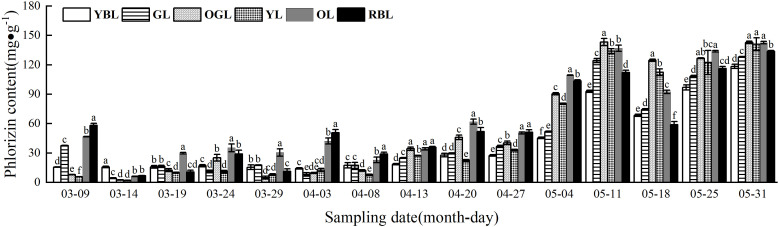
Phlorizin content of leaves of *Lithocarpus litseifolius* plants at developmental stage for different leaf colors.

The phlorizin content of *L. litseifolius* leaves exhibited a continuous upward trend during growth (**Figure 4**). The critical period for baby leaf growth and development was from 9 March to 27 April. During this stage, leaf growth was rapid, and leaf area became comparable to that of mature leaves. Meanwhile, phlorizin content rose from an average of 6.28 mg/g (14 March) to 39.88 mg/g by 27 April. It continued to increase after 27 April, reaching a maximum on 30 May. The values were: YBL (118.43 ± 2.13 mg/g), GL (128.12 ± 0.33 mg/g), OGL (143.87 ± 1.23 mg/g), YL (141.01 ± 6.44 mg/g), OL (142.69 ± 1.16 mg/g), and RBL (133.64 ± 1.08 mg/g)—approximately 7.57, 3.41, 18.21, 24.44, 3.05, and 2.30 times higher than germination levels, respectively. Among mature leaves, phlorizin content ranked as OGL > OL > YL > RBL > GL > YBL, with no significant differences between OGL, OL, and YL.

#### 3.3.3 Trilobatin content in leaves of different leaf colors of *L. litseifolius*

**Figure 5:**
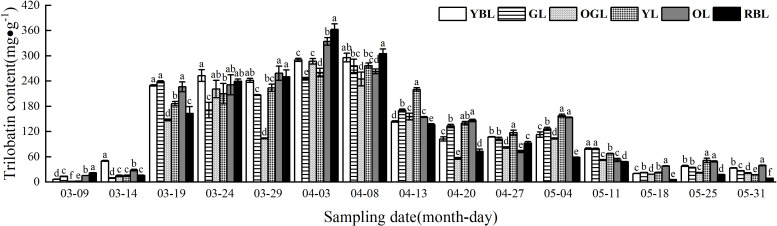
Trilobatin content of leaves of *Lithocarpus litseifolius* plants at developmental stage for different leaf colors.

Trilobatin levels in *L. litseifolius* followed an “ascending-declining-flat” trend (**Figure 5**). Synthesis began on 9 March, when the leaves first unfolded, with initial levels of 10.15 mg/g and 22.21 mg/g. By 19 March, levels surged to 198.68 mg/g—19.57 and 8.95 times higher than on 9 and 14 March, respectively. This coincided with a rapid growth phase, during which YBL, YL, OL, and RBL continued to increase, while GL and OGL briefly declined before rebounding. On 3 April, OGL, OL, and RBL reached their peak levels. The order of trilobatin content across leaf color types ranked as RBL (362.67 ± 13.50 mg/g) > OL (334.19 ± 8.95 mg/g) > YBL (290.19 ± 3.69 mg/g) > OGL (287.06 ± 6.66 mg/g) > YL (260.57 ± 9.72 mg/g) > GL (245.53 ± 3.10 mg/g). RBL and OL had significantly higher contents than the other leaf types, while YBL and OGL showed no significant difference. GL had the lowest level. On 8 April, trilobatin levels peaked in YBL, GL, and YL leaves, with the following order: RBL (305.24 ± 11.26 mg/g) > YBL (295.87 ± 10.20 mg/g) > YL (276.79 ± 6.07 mg/g) > GL (275.96 ± 15.88 mg/g) > OL (263.12 ± 5.60 mg/g) > OGL (245.18 ± 16.32 mg/g). RBL had the highest content and OGL the lowest, with the former 1.24 times higher than the latter. Trilobatin levels dropped sharply on 13 April and, by 18 May, had declined to 21.43 mg/g—a 92.26% decrease from 8 April—returning to early leaf development levels.

**Figure 6:**
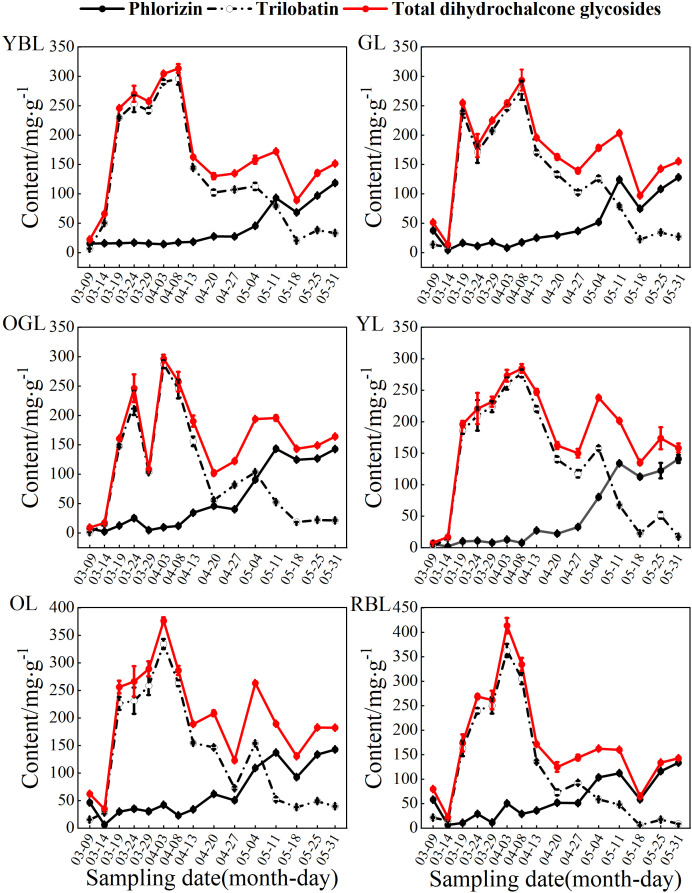
Comparison of phlorizin, trilobatin, and total dihydrochalcone glycosides contents in plants of various leaf colors.

Dihydrochalcone glycosides comprises phlorizin, its positional isomer trilobatin, and phloretin. Due to the extremely low content of phloretin, total dihydrochalcone glycosides content in this study was roughly estimated as the sum of phlorizin and trilobatin (Wang et al., 2023). Before 18 May, total dihydrochalcone glycosides was largely influenced by trilobatin, showing similar fluctuations. After 18 May, both total dihydrochalcone glycosides and phlorizin levels increased in tandem (**Figure 6**).

The synthesis of growth biochemicals began on 9 March, with total dihydrochalcone glycosides levels initially at their lowest. As trilobatin accumulated in the leaves, total dihydrochalcone glycosides content rose sharply, peaking during leaf development: OGL (296.69 mg/g), OL (376.39 mg/g), and RBL (413.47 mg/g) peaked on 3 April, while YBL (313.29 mg/g), GL (293.41 mg/g) and YL (273.17 mg/g) peaked on 8 April. On 29 March, OGL’s total dihydrochalcone glycosides content declined significantly, reflecting trilobatin’s influence. After 8 April, total dihydrochalcone glycosides levels decreased but rose again with phlorizin accumulation on 4 or 11 May, then dropped to their second-lowest point on 18 May as both trilobatin and phlorizin declined. After this, all six leaf color types underwent leathering. While trilobatin gradually declined, phlorizin increased, leading to a slight rebound in total dihydrochalcone glycosides.

### 3.4 Correlation analysis between leaf color and medicinal and food active ingredients

**Figure 7:**
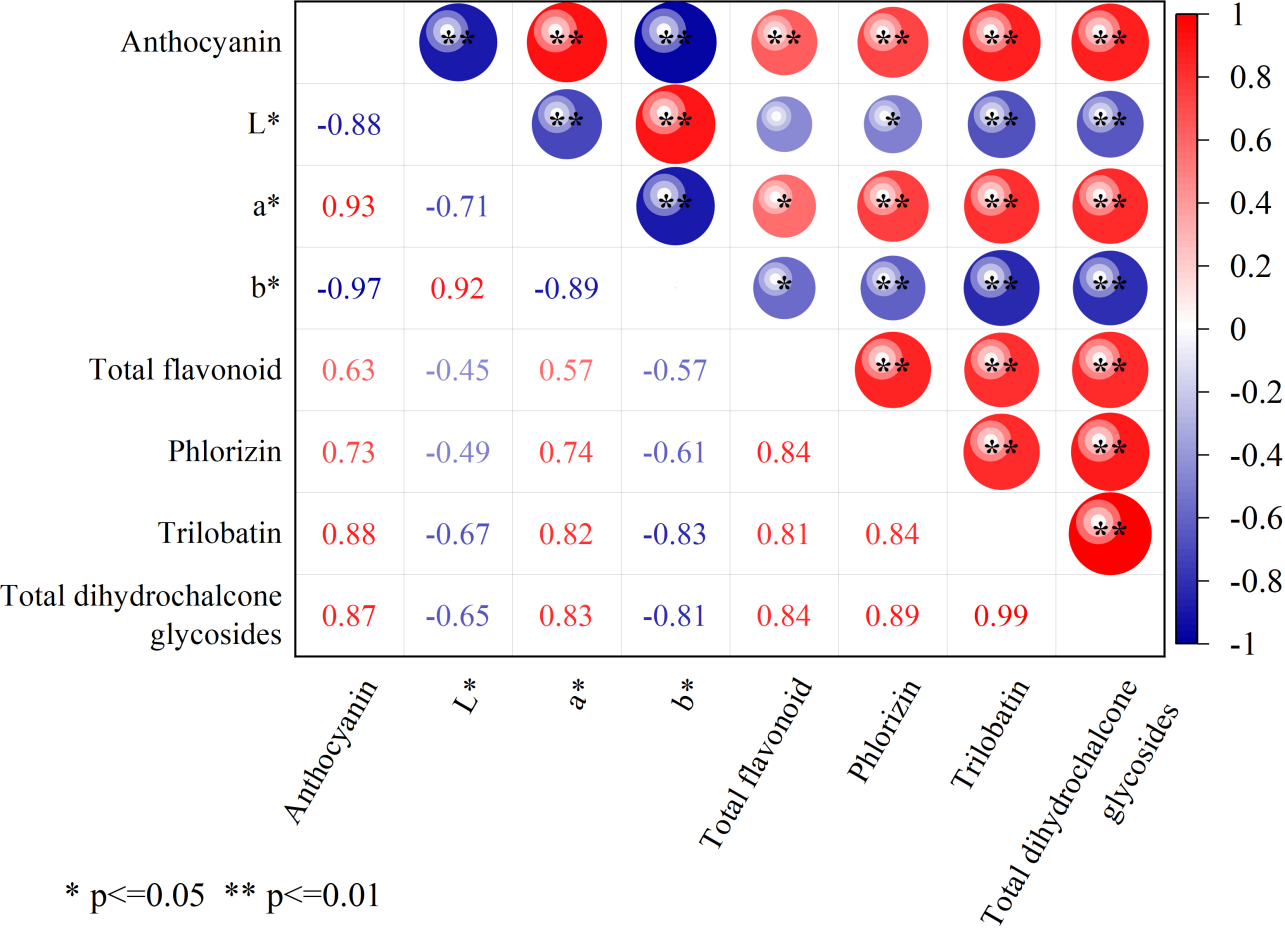
Correlation analysis between leaf color and the medicinal and food active ingredients. ** means extremely significant difference (P < 0.01), * means significant difference (P < 0.05).

*L. litseifolius* leaves are typically harvested at the end of March for tea production due to their high flavonoid and trilobatin content at this stage (Yang J. et al., 2018; Wei et al., 2020). This study analysed the relationship between leaf color and active medicinal and nutritional compounds at that time (**Figure 7**). Results indicated a significant correlation between flavonoid levels and color parameters a* and b*, with no such association observed for L*. However, flavonoid levels were also highly and positively correlated with anthocyanin levels. Phlorizin, trilobatin, and total dihydrochalcone glycosides all showed negative correlations with color values: phlorizin showed a significant negative correlation with L* (P < 0.05), while the other two showed highly significant negative correlations (P < 0.01). Conversely, all three compounds exhibited strong positive correlations with a* and anthocyanins (P < 0.01). These findings suggest that the a* (redness) value and anthocyanins of leaves were strongly correlated with the accumulation of active medicinal and nutritional ingredients, supporting the use of leaf color to estimate the key component content of *L. litseifolius*.

## 4 Discussion

### 4.1 Characteristics of medicinal and food active ingredient accumulation in *L. litseifolius* spring leaves

Plants exhibit different metabolite accumulation patterns at different growth stages or in different environments (Wang et al., 2018; Teixeira and Ng, 2017). These accumulation curves include linear, S-shaped, parabolic, fluctuating, and multi-peaked types. In this study, the total flavonoid content in *L. litseifolius* leaves showed a multi-peak pattern, with an overall increasing and then decreasing trend. When the leaves first spread, the total flavonoid content was very low, increased with growth, and peaked in late March/early April. The leaves then entered the maturity stage, and the content stabilized. During this period, three peaks in total flavonoid content were observed, with the first two significantly higher than the third. This may be due to the rapid leaf growth phase before 8 April, during which the plant is more vulnerable to environmental stresses (e.g., UV, pests, diseases), leading to increased synthesis of certain secondary metabolites (e.g., phenolics, flavonoids) to alleviate these stresses (Wang et al., 2025). In contrast, before the third peak, leaf growth was essentially complete. The young leaves had matured into thick, leathery green leaves, their defenses were relatively weaker, and flavonoid accumulation had declined. Similar patterns occur in other plants as well. For example, *Mitragyna* sp*eciosa* young leaves contain higher levels of medicinal alkaloids, which decrease as the leaves mature (Veeramohan et al., 2023). In *Scutellaria barbata*, total flavonoids and baicalin in stems and leaves are highest during peak growth and gradually decrease with flowering (Xu et al., 2018; Cheng et al., 2024). Similarly, the flavonoid content in stems and leaves of *Dendrobium officinale* peaks during the late growth stage (Wang et al., 2025).

Dihydrochalcone glycosides, the main flavonoid in *L. litseifolius* leaves, includes phlorizin, trilobatin, and phloretin. In this experiment, leaves germinated at the same time were sampled continuously, revealing alternating high and low levels of trilobatin and phlorizin in *L. litseifolius* leaves, consistent with previous studies (Wang Y. et al., 2019; Wang et al., 2016). During the rapid growth phase, the phlorizin content ranged from 6.28 mg/g to 39.88 mg/g. As the leaves matured, phlorizin increased and peaked in late May. Trilobatin content rose sharply during rapid growth, peaked in early April, then gradually declined after 11 May, and was nearly zero after maturity. Phlorizin and trilobatin are positional isomers of glycoside-bound flavonoids synthesized via the phenylalanine and flavonoid biosynthesis pathways (Ibdah et al., 2018; Gosch et al., 2010). Enzymes involved in trilobatin biosynthesis are likely expressed during rapid leaf growth, contributing to its accumulation. Elevated levels of phlorizin and its isomers, including trilobatin, are thought to sustain leaf morphology and physiological function by regulating photosynthesis and stress resistance (Dare et al., 2017; Zhou et al., 2019). Increased phlorizin biosynthesis has also been associated with enhanced photosynthetic carbon accumulation (Zhou et al., 2021; Wang et al., 2022). Zhou et al. (2021) demonstrated that silencing *MdUGT88F1* significantly disrupted phlorizin production and destabilize *MdGLK1/2* expression, leading to impaired chloroplast development, reduced chlorophyll biosynthesis, and weakened photosynthetic carbon fixation. In contrast, overexpression of *MdUGT88F1* increased phlorizin accumulation; although chloroplast development was not affected, the tricarboxylic acid (TCA) cycle was inhibited through metabolic competition, reducing carbon skeleton consumption during nitrogen assimilation and indirectly promoting sugar accumulation. Recent work further indicates that sustained phlorizin enrichment forms a stable carbon reservoir functioning as a metabolic “carbon sink,” thereby maintaining carbon homeostasis (Li et al., 2024). In *Medicago sativa*, nano-selenium treatment has likewise been reported to increase phlorizin levels and photosynthetic efficiency, with phlorizin showing synergistic regulation with starch and sucrose metabolic pathways (Sun et al., 2025).

### 4.2 Correlation of leaf color with flavonoid composition

Leaf color is closely linked to flavonoid composition and concentration, which are generally significantly higher in red, purple, or yellow leaves than in green leaves. (Yang T. et al., 2018) identified cyanidin as the predominant anthocyanin responsible for the purple-red coloration of young leaves in the crabapple cultivar ‘Indiamagic’ (*Malus* spp.). Similarly, variegated leaves of *Quercus gilva* contain higher flavonoid levels than non-variegated leaves (Lin et al., 2024). Leaf color in fresh tea leaves also influences processing suitability and final tea quality; purple leaves enriched in anthocyanins typically contain more bioactive compounds, underscoring leaf color as a reliable indicator of tea quality. Wang et al. (2020) investigated six locally cultivated varieties—large green leaf, small green leaf, large purple leaf, small purple leaf, green bud, and purple bud—and reported that the total flavonoid content of the small purple leaf variety (168.48 mg/g) was 3.28 times that of the large green leaf variety (51.32 mg/g). These results are consistent with our observations, which likewise showed higher total flavonoid levels in red-brown leaves.

A significant correlation has been observed between leaf color and flavonoid content. Flavonoids, including dihydrochalcone glycosides and anthocyanins, are synthesized through the phenylpropanoid pathway. In some cases, feedback inhibition within the flavonoid biosynthetic pathway or the downregulation of essential enzymes destabilizes the associated metabolic complex (Ueyama et al., 2002; Tanaka and Ohmiya, 2008; Ferreyra et al., 2012). Chalcone synthase (CHS) is regarded as the key enzyme in the biosynthesis of anthocyanins and dihydrochalcone glycosides (Ferrer et al., 2008). Yahyaa et al. (2017) showed that the three *CHS* genes in apple (*MdCHS1*, *MdCHS2*, and *MdCHS3*) exhibit no significant substrate specificity. Dare et al. (2017) confirmed that transcriptional disruption of these genes in transgenic apple lines leads to substantial reductions in both anthocyanins and dihydrochalcone glycosides. Moreover, regulatory factors such as the MBW complex exert coordinated control over both branches of the pathway and thereby influence leaf coloration. For instance, *MdMYC2*, a bHLH transcription factor, has been reported to enhance anthocyanin accumulation by upregulating flavonoid biosynthetic genes, including *MdDFR*, *MdUF3GT*, *MdF3H*, and *MdCHS*. While this research emphasizes anthocyanins, it is important to note that dihydrochalcone glycosides utilize the same upstream CHS gene as anthocyanins in apples. Therefore, it is proposed that this complex may also enhance the accumulation of dihydrochalcone glycosides by upregulating the *MdCHS* gene (An et al., 2016). Conversely, the photosynthetic structures of newly formed leaves remain immature, and the synthesis of flavonoids mitigates photo-oxidative stress by providing photoprotective and antioxidant functions, including excess light absorption and reactive oxygen species (ROS) scavenging (Yu et al., 2020; Narbona et al., 2018; Xiao et al., 2017). Gaucher et al. (2013) reported that dihydrochalcone glycosides efficiently remove excessive ROS and filter ultraviolet radiation, thus conferring strong light protection to apple plants. Additionally, Zhou et al. (2021) found that apple plants overexpressing MdUGT88F1—an essential gene for phlorizin synthesis—accumulate higher levels of both phlorizin and anthocyanins. This environmentally driven mechanism, described as “physiological functional requirement → metabolite accumulation → phenotypic color expression” has been validated extensively (Xie et al., 2023; Cui et al., 2023).

## 5 Conclusions

The results showed that while the changing patterns of medicinal and food-active ingredients in the six leaf-colored spring leaves of *L. litseifolius* were consistent, their contents varied. Trilobatin peaked in April, when leaves grow rapidly. These tender leaves taste best in tea, so harvesting is recommended at this stage. Phlorizin peaked on 31 May, suggesting that leaves should be collected at full maturity for pharmaceutical use. Flavonoid content was higher in RBL and OL compared to other leaf colors. Furthermore, a correlation analysis was conducted between leaf flavonoid content and leaf color. This study revealed a significant positive correlation between redness (a*), anthocyanin content, and total flavonoid levels in the leaves of *L. litseifolius*. This relationship suggests that flavonoid accumulation in *L. litseifolius* can be estimated indirectly through visual assessment of leaf coloration and intensity. Accordingly, the study establishes a non-destructive visual screening approach that is expected to expedite the breeding and cultivation of high-value *L. litseifolius* varieties.

## Data Availability

The original contributions presented in the study are included in the article/**Supplementary Material**. Further inquiries can be directed to the corresponding author.
