# Supplementary material for: Leaf color as an indicator of the accumulation pattern of pharmaco-nutritional compounds in Lithocarpus litseifolius spring foliage
Source: Front Plant Sci. 2025 Dec 10;16:1726934. doi: 10.3389/fpls.2025.1726934 (PMC12729113; doi:10.3389/fpls.2025.1726934)
Supplement: Supplementary file 1 [file Table1.docx]

Table.1 Statistics on the growth trends of branches and leaves of *Lithocarpus litseifolius* with different leaf colours

| Materials | Observation date  (month-day) | Characteristic indicators | | | | |
| --- | --- | --- | --- | --- | --- | --- |
|  |  | New branch length  (mm) | Number of leaves on new branch | Leaf length (cm) | Leaf width (cm) | Leaf area  (cm^2^) |
| YBL | 03-09 | 15.72 | 6.48 | 0.75 | 0.24 | 0.04 |
|  | 03-14 | 46.78 | 8.97 | 1.69 | 0.48 | 0.24 |
|  | 03-19 | 80.48 | 9.90 | 2.38 | 0.84 | 0.87 |
|  | 03-24 | 117.11 | 10.97 | 3.59 | 1.04 | 1.76 |
|  | 04-03 | 131.84 | 11.30 | 4.45 | 1.32 | 3.04 |
|  | 04-08 | 141.05 | 11.53 | 5.26 | 1.64 | 4.70 |
|  | 04-13 | 157.26 | 11.57 | 7.23 | 2.35 | 9.93 |
|  | 04-20 | 161.08 | 11.63 | 9.35 | 3.33 | 19.03 |
|  | 04-27 | 167.86 | 11.77 | 10.06 | 4.05 | 24.45 |
|  | 05-04 | 171.28 | 11.93 | 10.31 | 4.27 | 27.16 |
|  | 05-11 | 176.42 | 12.00 | 10.75 | 4.43 | 30.43 |
|  | 05-18 | 180.22 | 12.20 | 11.22 | 4.64 | 33.28 |
|  | 05-25 | 181.61 | 12.20 | 11.42 | 4.83 | 34.51 |
|  | 05-31 | 182.03 | 12.20 | 11.54 | 4.87 | 34.85 |
| GL | 03-09 | 22.57 | 7.80 | 1.10 | 0.35 | 0.10 |
|  | 03-14 | 53.23 | 8.07 | 1.95 | 0.65 | 0.65 |
|  | 03-19 | 76.58 | 8.40 | 3.05 | 0.96 | 1.70 |
|  | 03-24 | 99.54 | 9.13 | 4.08 | 1.36 | 3.43 |
|  | 04-03 | 105.35 | 9.13 | 5.08 | 1.67 | 5.37 |
|  | 04-08 | 110.58 | 9.03 | 6.18 | 2.14 | 8.52 |
|  | 04-13 | 112.85 | 9.10 | 8.12 | 2.94 | 15.13 |
|  | 04-20 | 113.36 | 9.17 | 9.99 | 3.77 | 23.64 |
|  | 04-27 | 114.88 | 9.17 | 10.28 | 3.97 | 24.69 |
|  | 05-04 | 116.08 | 9.40 | 10.66 | 4.15 | 27.62 |
|  | 05-11 | 117.75 | 9.53 | 11.17 | 4.26 | 30.00 |
|  | 05-18 | 119.49 | 9.60 | 11.50 | 4.41 | 32.24 |
|  | 05-25 | 121.34 | 9.77 | 11.93 | 4.60 | 34.13 |
|  | 05-31 | 124.52 | 9.90 | 12.11 | 4.74 | 35.19 |
| OGL | 03-09 | 15.37 | 6.37 | 0.73 | 0.23 | 0.08 |
|  | 03-14 | 51.88 | 8.43 | 1.90 | 0.52 | 0.51 |
|  | 03-19 | 82.34 | 9.30 | 3.03 | 0.96 | 1.76 |
|  | 03-24 | 108.49 | 10.27 | 4.12 | 1.18 | 2.82 |
|  | 04-03 | 116.30 | 10.53 | 4.83 | 1.46 | 4.21 |
|  | 04-08 | 124.44 | 10.30 | 6.46 | 1.97 | 7.81 |
|  | 04-13 | 127.33 | 10.23 | 8.48 | 2.90 | 15.46 |
|  | 04-20 | 130.90 | 10.23 | 10.47 | 3.85 | 25.77 |
|  | 04-27 | 134.24 | 10.23 | 10.58 | 4.11 | 27.89 |
|  | 05-04 | 137.92 | 10.47 | 10.77 | 4.38 | 30.49 |
|  | 05-11 | 139.89 | 10.73 | 11.14 | 4.48 | 32.28 |
|  | 05-18 | 141.90 | 10.90 | 11.57 | 4.62 | 34.20 |
|  | 05-25 | 143.64 | 10.90 | 11.76 | 4.76 | 35.46 |
|  | 05-31 | 145.11 | 11.00 | 11.88 | 4.90 | 37.02 |
| YL | 03-09 | 12.76 | 5.57 | 0.70 | 0.20 | 0.09 |
|  | 03-14 | 49.35 | 9.23 | 1.79 | 0.47 | 0.53 |
|  | 03-19 | 82.48 | 9.27 | 2.97 | 0.83 | 1.46 |
|  | 03-24 | 111.69 | 9.93 | 4.09 | 1.11 | 2.87 |
|  | 04-03 | 123.97 | 10.50 | 4.92 | 1.37 | 4.25 |
|  | 04-08 | 130.98 | 10.60 | 6.09 | 1.75 | 6.76 |
|  | 04-13 | 135.68 | 10.73 | 8.46 | 2.54 | 13.57 |
|  | 04-20 | 139.89 | 10.97 | 10.88 | 3.71 | 25.48 |
|  | 04-27 | 144.14 | 11.17 | 11.03 | 4.09 | 28.20 |
|  | 05-04 | 147.69 | 11.40 | 11.29 | 4.37 | 31.14 |
|  | 05-11 | 151.11 | 11.63 | 11.70 | 4.60 | 33.29 |
|  | 05-18 | 154.88 | 11.83 | 12.15 | 4.77 | 35.55 |
|  | 05-25 | 156.89 | 12.00 | 12.21 | 5.03 | 38.18 |
|  | 05-31 | 159.33 | 12.07 | 12.50 | 5.10 | 39.86 |
| OL | 03-09 | 21.24 | 6.57 | 1.01 | 0.29 | 0.08 |
|  | 03-14 | 53.18 | 6.70 | 2.19 | 0.65 | 0.78 |
|  | 03-19 | 75.72 | 6.83 | 3.67 | 1.14 | 2.54 |
|  | 03-24 | 96.90 | 7.43 | 5.18 | 1.56 | 5.04 |
|  | 04-03 | 106.04 | 7.50 | 6.17 | 1.90 | 7.47 |
|  | 04-08 | 111.45 | 7.60 | 7.95 | 2.48 | 12.67 |
|  | 04-13 | 122.19 | 7.87 | 9.98 | 3.36 | 21.51 |
|  | 04-20 | 132.89 | 8.23 | 11.71 | 4.17 | 30.77 |
|  | 04-27 | 141.43 | 8.60 | 12.19 | 4.38 | 33.73 |
|  | 05-04 | 149.77 | 9.07 | 12.75 | 4.52 | 36.26 |
|  | 05-11 | 152.87 | 9.40 | 13.25 | 4.75 | 40.35 |
|  | 05-18 | 158.78 | 9.83 | 13.76 | 4.94 | 42.49 |
|  | 05-25 | 164.38 | 10.10 | 14.29 | 5.12 | 45.26 |
|  | 05-31 | 168.86 | 10.17 | 14.42 | 5.12 | 45.54 |
| RBL | 03-09 | 25.33 | 8.50 | 1.14 | 0.30 | 0.22 |
|  | 03-14 | 83.07 | 8.67 | 2.99 | 0.79 | 1.45 |
|  | 03-19 | 104.74 | 8.77 | 4.14 | 1.20 | 2.95 |
|  | 03-24 | 118.07 | 9.50 | 5.68 | 1.51 | 5.30 |
|  | 04-03 | 132.03 | 9.80 | 7.18 | 1.97 | 8.80 |
|  | 04-08 | 143.40 | 10.07 | 8.81 | 2.50 | 13.55 |
|  | 04-13 | 149.66 | 10.23 | 10.98 | 3.45 | 23.23 |
|  | 04-20 | 153.49 | 10.33 | 12.99 | 4.28 | 33.74 |
|  | 04-27 | 158.94 | 10.53 | 13.31 | 4.55 | 36.74 |
|  | 05-04 | 165.06 | 10.67 | 13.52 | 4.80 | 39.25 |
|  | 05-11 | 170.36 | 10.93 | 13.84 | 4.99 | 42.37 |
|  | 05-18 | 175.96 | 11.20 | 14.15 | 5.17 | 44.89 |
|  | 05-25 | 183.29 | 11.55 | 14.58 | 5.38 | 48.11 |
|  | 05-31 | 187.20 | 11.73 | 14.93 | 5.48 | 50.53 |

Note: The values in the table represent the average of 30 observed branches.
